# Stability Analysis of Thin-Walled Perforated Composite Columns Using Finite Element Method

**DOI:** 10.3390/ma15248919

**Published:** 2022-12-13

**Authors:** Katarzyna Falkowicz

**Affiliations:** Department of Machine Design and Mechatronics, Faculty of Mechanical Engineering, Lublin University of Technology, Nadbystrzycka 36, 20-618 Lublin, Poland; k.falkowicz@pollub.pl

**Keywords:** FEM, stability, linear and nonlinear analysis, holes, failure, composites, thin-walled structure

## Abstract

Open holes or cut-outs have been commonly used in composite structures for various engineering purposes. Those elements often demand perforation especially for weight reduction and to ease maintenance and servicing operations, for example, in aircraft wing ribs. This work presents a numerical study of the stability behavior of composite perforated columns subjected to a compressive load. Profiles were made of CFRP laminate and weakened by three types of cut-out. Four parameters, spacing ratio *S*/*D*_0_, opening ratio *D*/*D*_0_, hole shape and arrangement of layers, were selected to check their effect on the buckling load and postbuckling behavior of the tested channel profiles. To carry out the numerical analysis, the Abaqus software was used. The results obtained during the analysis helped to identify the best combination of tested parameters to obtain the highest critical load. The performed analysis show that the columns’ behavior is sensitive to configuration of composite, opening ratio and hole shape.

## 1. Introduction

Thin-walled structures belong to the load-carrying elements characterized by high stiffness and strength while maintaining low weight [1,2,3,4] and that allows designers to have freedom in shaping the construction form. Thanks to the mentioned properties, these kinds of structures have wide application, where the low weight is important, i.e., aerospace and automotive sectors and civil engineering [1,5,6]. Unfortunately, as in any thin-walled elements, they have important disadvantages such as loss of stability when they are compressed or sheared [7,8,9,10]. However, they have one of the most important features which is the possibility of working in the postcritical state when the buckling of a thin-walled element is local and elastic [11,12,13,14,15,16]. In the worldwide available literature, more studies about linear and nonlinear problems of thin-walled structures’ stability can be found [17,18,19]. Moreover, thin-walled elements often demand perforation for weight reduction and to ease servicing and maintenance operations, for example, in aircraft wing ribs. These perforations cause a redistribution of stresses in the structure that may change the strength of the structural element and the elastic stiffness. The buckling behavior of structural perforated profiles is significantly influenced by the size, shape, location or number of perforations. Previous investigations on composite plates [20,21,22,23,24] have shown the significant effect of the geometric parameters and shape of holes, as well as the composite configuration, on the stability behavior. Therefore, it seems imperative to investigate the influence of the abovementioned parameters on thin-walled perforated profiles’ buckling behavior.

**Figure 1:**
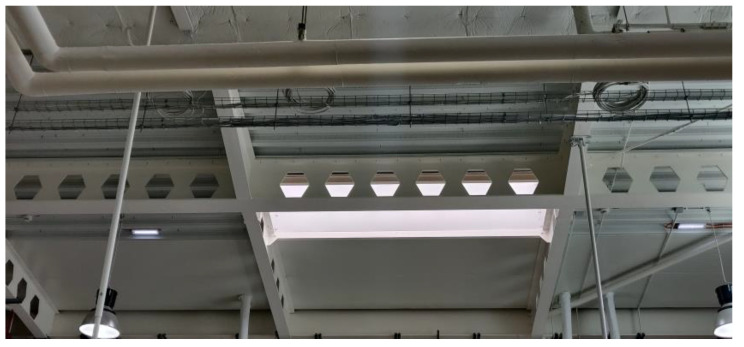
Example of application of perforated elements.

Thin-walled channel profiles are currently widely used and often weakened by holes to reduce their volume. An example of such elements are perforated profiles used, among others, for shelves, balustrades, etc. In Figure 1, an example of popular design solutions based on the use of openwork beams as load-carrying structures is shown.

In the literature, there are a lot of investigations about the design and behavior of columns with different kinds of cross-sections made of composite [4,25,26] or traditional materials [27,28]. Moreover, in the available literature there are some examinations where the authors tested elements with different kinds of hole shapes [29,30,31]. However, the obtained results showed that most studies of the literature concern structures without holes, or with holes, but made of traditional materials. However, the knowledge about buckling behavior of perforated composite columns is insufficient. Furthermore, there are no specific standards for composite perforated profiles such as in case of perforated profilers made of steel or aluminum.

The load-bearing capacity of composite profiles, due to wide usage of composite elements in a thin plate form, is very important. Furthermore, different kinds of cut-outs are very often made as a part of the project. This makes the information about the buckling and postbuckling behavior of composite profiles weakened by holes necessary.

The idea of this work is based on the study carried out by Khazaal et al. [32], who tested aluminum alloy profiles subjected to compression. To optimize the results and to check which parameter has the most influence on the buckling behavior of the aluminum thin-walled elements, they investigated three shapes of hole: rectangular, circular and hexagonal. A very similar investigation was described in paper [33], where the authors took under consideration profiles made from GFRP composite. It is worth adding here that, in previous articles [34,35], the authors tested composite profiles with different kinds of cross-section but without perforation. Therefore, the abovementioned studies together with [32,33] have ignited motivation for the presented study, hence contributing towards the knowledge on thin-walled composite structures with holes.

This paper includes the linear and nonlinear analysis of compressed, thin-walled, perforated profiles with a channel cross-section. Numerical calculations were carried out with the commercial Abaqus program using the finite element method (FEM). The effect of parameters such as hole shapes, spacing ratio, opening ratio and arrangement of layers’ layout on the buckling and postbuckling behavior of the perforated channel cross-section profiles was investigated. Moreover, obtained results were compared with unperforated profiles which were tested in previous works [9,35,36]. It should be added here that the tested columns were made of CFRP composite material, and previous research has been carried out on unperforated profiles or on perforated profiles but made from classic materials, including paper [31], where the authors tested perforated profiles made of GFRP composite. It should also be mentioned here that there are no specific standards for the perforation of composite profiles. Furthermore, columns made of CFRP composite have not been taken under this kind of consideration. Therefore, it was a good motivation for conducting the analysis presented in this paper.

## 2. Research Subject and Methodology

**Figure 2:**
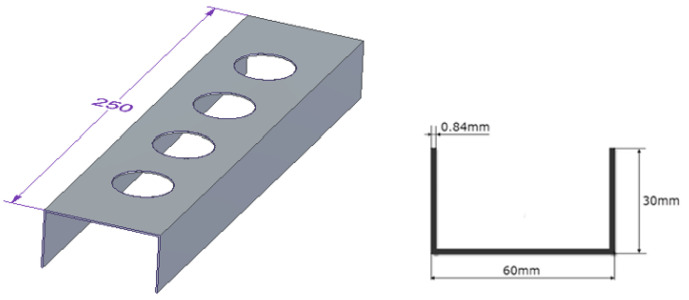
Geometric parameters of the tested profile.

The research subjects were thin-walled composite profiles with a C cross-section subjected to axial compression. The geometric parameters of tested profiles are shown in Figure 2 (length—250 mm, the web—60 mm, the profile wall h = 30 mm). The tested columns consisted of 8 plies symmetric to the laminate mid-plane. Each ply of profiles had a thickness of 0.105 mm and the total thickness of the wall was 0.84 mm. The profiles were made of CFRP composite, whose mechanical properties are presented in Table 1.

**Figure 3:**
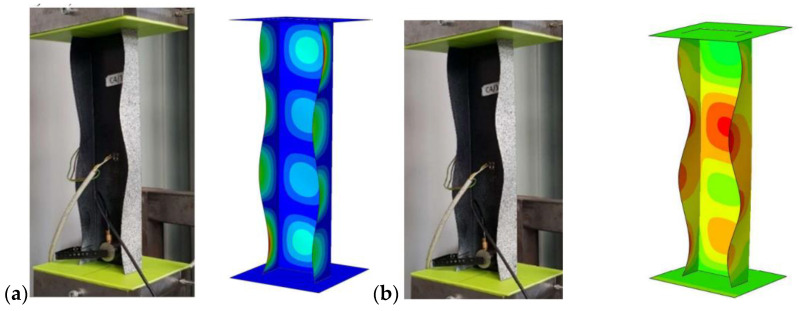
(**a**) Form of buckling, (**b**) form of postcritical deformation.

According to the previous research [7,36,37], four configurations of composite were chosen for the analysis: P1: [0/45/−45/90]s, P2: [45/−45/90/0]s, P3: [90/−45/45/0]s, P4: [90/0/90/0]s. The profiles were compared with profiles without cut-outs, which were also tested experimentally and numerically in previous works [34,37,38]. The exemplary obtained buckling and postcritical deformation forms are shown in Figure 3.

The results presented above became a reference point for the research performed and presented in this paper. The current paper focuses on the behavior of channel cross-section profiles weakened by holes and subjected to axial compression. This work does not concern the in-depth presentation of the experimental results, it focuses on the numerical analysis, but this is the start for further experimental research.

**Figure 4:**
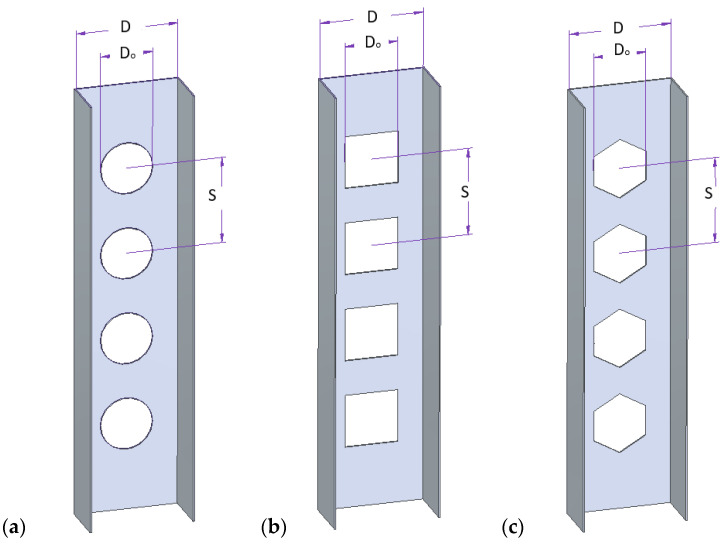
Shapes and dimensions of holes: (**a**) circle, (**b**) square, (**c**) hexagonal.

In this work, according to [32,33], three shapes of holes (circular, square, hexagonal) made in the profile web were designed (Figure 4). The overall geometric parameters were constant. In [32], the authors tested aluminum profiles where the geometry of web holes must be within the given ranges of Eurocode (Equations (1) and (2)) in order to prevent any unwanted failures such as cracks between holes, and to achieve a maximum possible reduction in weight.

1.25 < *D*/*D*_0_ < 1.75 (1)

1.08 < *S*/*D*_0_ < 1.5 (2)

where: *D*—width of profile web; *D*_0_—diameter/width of hole; *S*—distance between holes; *D*/*D*_0_—opening ratio; *S*/*D*_0_—spacing ratio.

For composite profiles, this kind of Eurocode does not exist but it was a good reason to take those equations under consideration and perform numerical analysis for composite columns with a channel section. The above equations were used in [33] to analyze perforated profiles made of GFRP composite.

Three parameters, opening ratio (*D*/*D*_0_), spacing ratio (S/*D*_0_) and hole shape, were taken under consideration, and their influence on buckling and postbuckling behavior was measured. The opening ratio (*D*/*D*_0_) and spacing ratio (S/*D*_0_) were selected according to Equations (1) and (2). Parameters which were outside the bounds of the above equations were also taken into consideration. The parameters and levels are presented in Table 2.

## 3. Numerical Analysis

The numerical calculations were carried out in the Abaqus program with the finite element method, which has a very wide range of applications [39,40]. Analysis was performed in two stages. The first stage was the linear stability analysis, based on the solution of the following generalized eigenvalue problem:

|[K] + λi [H]| = 0(3)

where: [K]—structural stiffness matrix, λi—the i-th eigenvalue, [H]—stress stiffness matrix.

The critical state of the structure was described by a linear model using the minimum total potential energy principle. Equation (3) presents mathematical notation of the loss of stability phenomenon. The scope of numerical simulations involved describing the critical state of the structure and influence of shape and parameters of cut-outs on critical force.

The second stage of analysis tackled the nonlinear stability problem, based on the incremental iterative Newton–Raphson method. This analysis allowed us to determine the equilibrium path for the structure. The calculations were performed until failure initiation of first layer according to the Tsai–Wu criterion [41].

**Figure 5:**
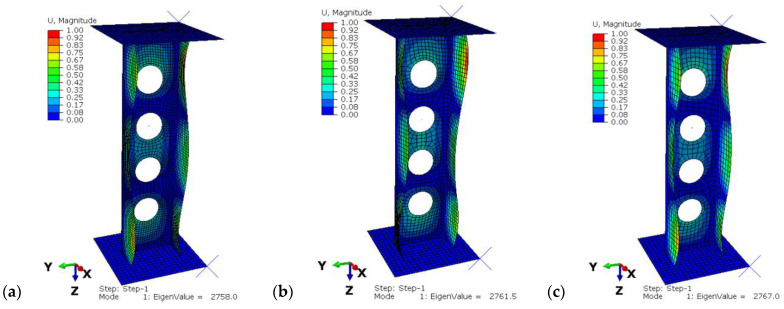
The results from convergence test: (**a**) mesh size = 3 mm, (**b**) mesh size = 4 mm, (**c**) mesh size = 5 mm.

The discrete model was prepared by using 8-node shell elements (S8R) of second order with reduced integration. The structure of the composite material was defined depending the thickness of the finite element. Mesh density, which was used for the composite profiles, equaled 4 mm and was selected as the optimal density according to the previous analyses on unperforated profiles with channel cross-sections [32]. The value of buckling load obtained for mesh size 4 mm was the closest to the buckling load obtained by experiment and the percentage error was around 2%. However, additionally, the convergence was studied for three different mesh sizes: 3 mm, 4 mm and 5 mm. The results of tests for profiles with P2 lay-up are presented in Figure 5. Obtained results showed that the mesh size has no significant influence on buckling behavior. Furthermore, denser mesh lengthens the processing time. Similar conclusions were presented in [34,40,42].

**Figure 6:**
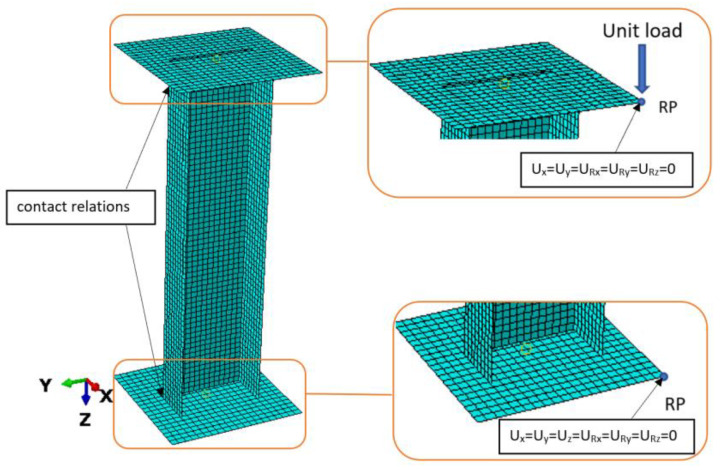
Boundary conditions of tested profile.

The boundary conditions of the tested model are presented in Figure 6. The model consisted of two rigid plates as a support. The upper plate had the ability to move along the *Z*-axis (compressive force direction) and the lower plate was fully fixed. The boundary conditions were defined in a created reference point (RP), connected to plates. Between the plates and composite profile, a contact interaction was introduced with a friction coefficient of 0.2.

## 4. Results and Discussion

**Figure 7:**
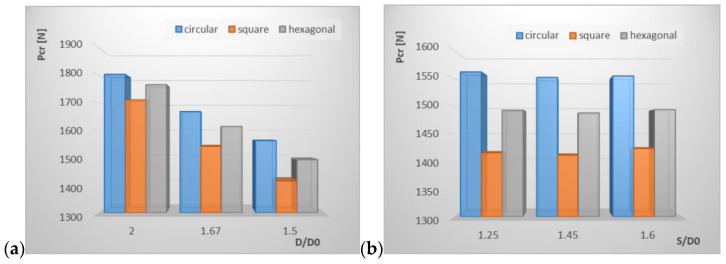
The effect of opening ratio (**a**) and spacing ratio (**b**) on buckling load for three different hole shapes.

In this section, the results from the performed numerical analysis are presented. Table 3 shows the values of critical forces for all tested cases with different kinds of holes shape. The below results present the buckling load for the P1 configuration of the composite. Ratios which exceed ranges of Equations (2) and (3) are marked in red. In the last column is the percentage difference between critical force for unperforated profiles and for perforated tested profiles. In Figure 7, for better visualization, the effect of spacing and opening ratio on the buckling load for three different hole shapes is presented.

The presented results from FE analysis show that the introduction of perforation leads to the load decrease. The circular holes caused a smaller decrease of critical load and for all tested cases it was in the range of 17 ÷ 28.48%. For square holes, this range was 21.30 ÷ 35.06% and for the hexagonal holes it was 18.76 ÷ 31.54%. It can be observed that when increasing the opening ratio, the buckling load also increased (Figure 7a), whereas the effect of the spacing ratio (Figure 7b) did not show any significant influence on the value of critical load. Those observations confirm the results obtained in [33] for GFRP composite profiles.

It can be observed that among all tested samples with different kinds of hole shapes, the circular holes had the greatest impact on the buckling load with opening ratio *D*/*D*_0_ = 2 and with spacing ratio *S*/*D*_0_ = 1.67. In [33], the best ultimate strength was also seen for the circular shape, while in [32] the best results were for the hexagonal shape.

**Figure 8:**
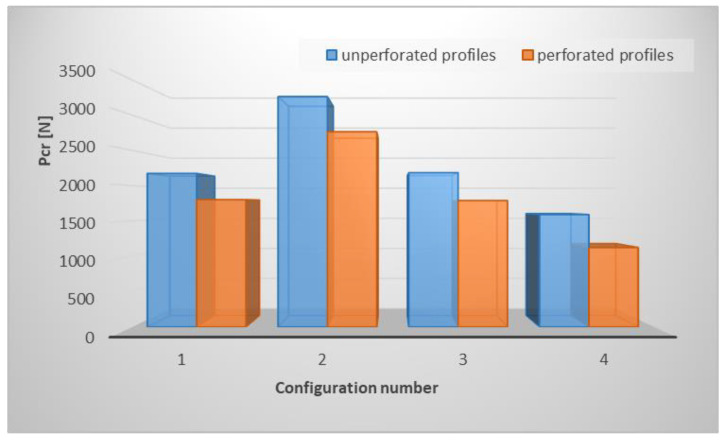
Effect of opening ratio and space ratio on critical buckling load for different hole shapes.

In this section, the results of the lay-up effect on the buckling behavior of the tested profiles are presented. Table 4 shows the obtained values of critical loads for perforated and unperforated profiles, for four different composite lay-ups. For this analysis, the profile with a circular hole shape with the best results of parameters, which were tested in the first part of the analysis, was chosen. Figure 8 additionally presents the same results but in the form of a column chart.

**Figure 9:**
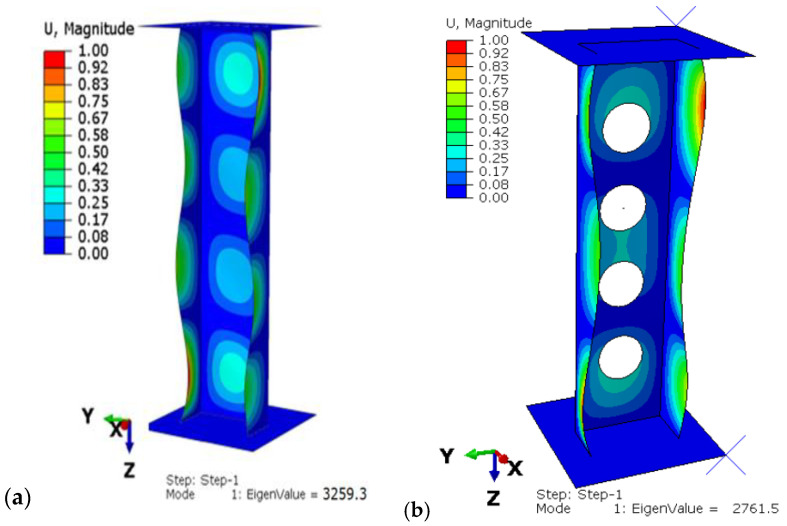
Buckling simulation results for the P2 lay-up: (**a**) unperforated profile, (**b**) profile with circular holes.

It can be observed that the laminate lay-up has a significant effect on buckling load. Furthermore, the weakest configuration is P4 and the difference between buckling load for unperforated and perforated profiles for this configuration is more than 20%, compared to the other three configurations where the difference was less than 20%. The highest critical load was obtained for the P2 lay-up. This is ascribable to the fact that, for P2, most outer layers are at an angle of 45 degrees, whereas for P4 the lay-up consists of a few layers at an angle of 90 degrees, which reduces the overall structural stiffness. In all cases, the shape of the buckling mode maintained its symmetrical character with respect to the symmetry plane of the C-profile (Figure 9). However, for almost all lay-ups, the local buckling of the web and shelf was characterized by different numbers of half-waves. The number of half-waves for the perforated profiles was lower by one or two compared to the unperforated profiles.

The place of maximum deformation was also different: for the P1 and P2 configurations, it was on the shelf closest to the rigid plate and for the P3 and P4 configuration it was in the middle of the profile shelf.

**Figure 10:**
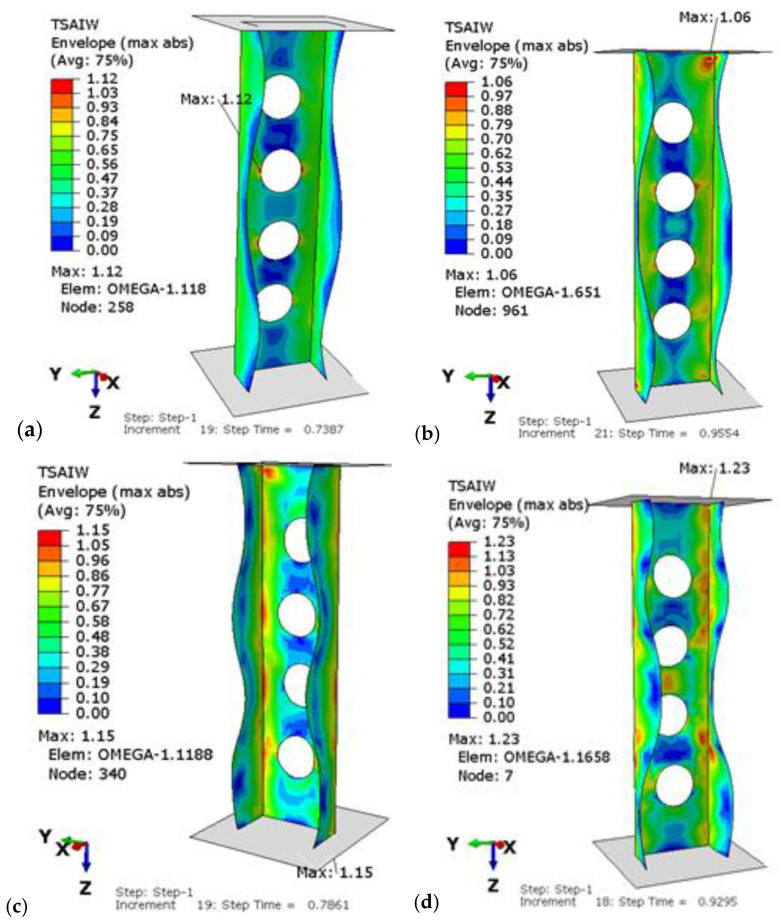
Tsai–Wu critical parameter map with the obtained form of loss of stability for the configurations: (**a**) P1, (**b**) P2, (**c**) P3, (**d**) P4.

**Figure 11:**
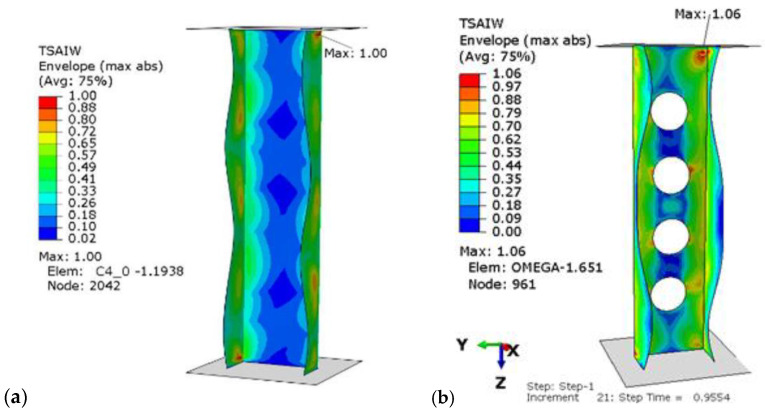
Tsai–Wu critical parameter map with the obtained form of loss of stability for the P2 configuration for: (**a**) unperforated, (**b**) perforated profile.

In the second part of the research, the nonlinear stability of the structure in the postbuckling range was measured. The aim of the analysis was to investigate the profile behavior in the postcritical state with the implemented lower buckling mode, which was determined in the first part of the tests. During the loading of the structure, a deepening form of laminate buckling was observed. The load-carrying capacity of tested profiles was determined by the Tsai–Wu failure criterion. This criterion was successfully used to determine the failure load of a compressed thin-walled composite plate element weakened by holes and columns with complex cross-sectional shapes in [21,43,44]. Figure 10 shows a designated example map of the Tsai–Wu criterion, where areas in which damage initiation of the first composite layer may occur, corresponding to the achievement of the value of 1 critical parameter, determined according to the above criterion, are presented. Moreover, in Figure 11, failure maps for perforated and for unperforated profiles for the chosen P2 configuration are compared.

**Figure 12:**
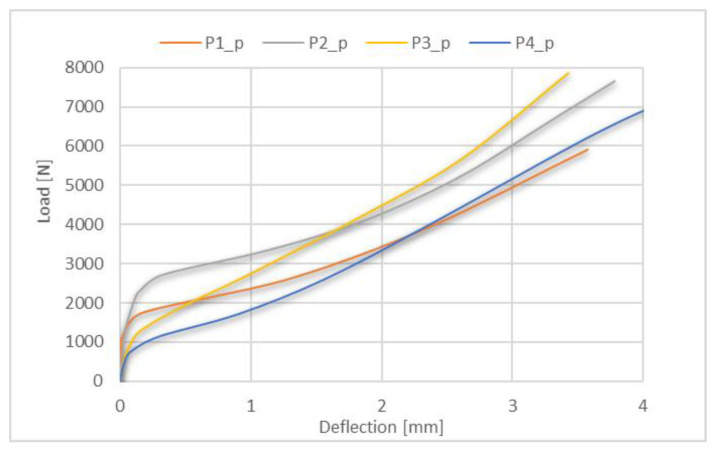
Postbuckling equilibrium paths for perforated profiles with all tested lay-ups.

**Figure 13:**
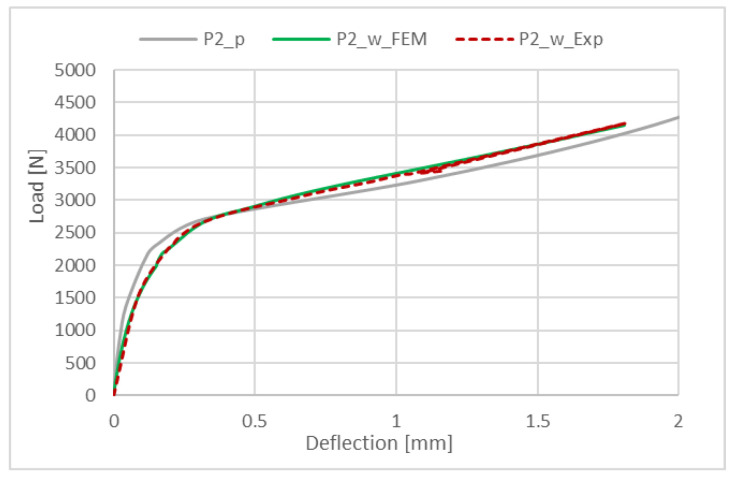
Postbuckling equilibrium paths for perforated profiles with P2 lay-up and for unperforated profiles with P2 lay-up (results from numerical and experimental analysis).

It can be observed that the most critical area is the corner near to the end of the cross-section. Moreover, the introduction of perforation did not change this localization. Figure 12 additionally presents the obtained equilibrium paths for the perforated profiles for the four considered lay-ups. Moreover, in Figure 13, the equilibrium paths are compared for the perforated and unperforated profiles with P2 lay-up.

The obtained results show that for all of the tested profiles, the postbuckling equilibrium paths are stable. In addition, the postbuckling behavior of perforated and unperforated profiles is similar at the beginning. However, with the deepening of the deflection, the differences between the equilibrium paths increase. The highest deflection at failure was observed for the P4 lay-up and the lowest one for the P2 lay-up. Furthermore, the equilibrium path for the P2 laminate lay-up seems to be the one with the highest stiffness.

## 5. Conclusions

In this study, the linear and nonlinear analysis of perforated composite profiles with a C cross-section subjected to axial compression was performed. The effect of three hole shapes, opening ratio *D*/*D*_0_, the spacing ratio *S*/*D*_0_ and composite arrangement of layout on buckling behavior of perforated profiles was studied. The obtained results were compared with profiles without holes. On the basis of the obtained results, some conclusions can be drawn:

➢ The introduction of perforations caused a decrease in the buckling loads: 21.30 ÷ 35.06% for the square shape, 17 ÷ 28.48% for the circular shape and 18.76 ÷ 31.54% for the hexagonal shape.
➢ The obtained results show that the shape of holes, opening ratio and arrangement of laminate layers have the greatest impact on the value of buckling load, whereas the spacing ratio has no significant influence on the buckling load.
➢ The circular holes with *D*/*D*_0_ = 2 and *S*/*D*_0_ = 1.67 gave the highest value of critical force.
➢ The highest critical load was obtained for the P2 lay-up, while the lowest one for the P4 lay-up.
➢ The introduced perforation caused not only a decrease in the critical load value, but also a change in the buckling form. The decrease in the critical load for all tested configurations was: 17.4% for P1, 15.27% for P2, 18.08% for P3 and 29.32% for P4.

The presented results allow us to expand our knowledge about the design of thin-walled composite structures weakened by holes with potential significance for practical applications. However, to confirm the obtained numerical results, a deeper analysis of nonlinear stability conditions and experimental validation are necessary.

## Figures and Tables

**Table 1:** Mechanical properties of a CFRP lamina.

Young’s Modulus [MPa]		Shear Modulus [MPa]	Poisson’s Ratio	Tensile Strength [MPa]		Shear Strength [MPa]	Compression Strength [MPa]	
**E_1_** **0°**	**E_2_** **90°**	**G_1,2_**	**V_12_**	**F_TU1_** **0°**	**F_TU2_** **90°**	**F_SU_** **45°**	**F_CU1_** **0°**	**F_CU2_** **90°**
143,530	5826	3845	0.36	2221	49	83.5	641	114

**Table 2:** Parameters and levels.

Parameter	Levels		
	1	2	3
Shapes of holes	Circular	Square	Hexagonal
*D*/*D*_0_*(S* = *const)*	2	1.67	1.5
*S*/*D*_0_*(D*_0_ = *const)*	1.25	1.45	1.6

**Table 3:** Buckling load value for all tested cases for P1 composite configuration.

No.	Hole Shape	Opening Ratio *D*/*D*_0_	Spacing Ratio *S*/*D*_0_	Pcr [N]	Difference [%]
1	Without holes	---	---	2172.4	-
2	Circular	2	1.67	1802.3	17.04
3	Square			1709.6	21.30
4	Hexagonal			1764.8	18.76
5	Circular	1.67	1.39	1667.3	23.25
6	Square			1541	29.06
7	Hexagonal			1612.9	25.75
8	Circular	1.5	1.25	1561.3	28.13
9	Square			1415.2	34.85
10	Hexagonal			1492.1	31.32
11	Circular	1.5	1.45	1551.3	28.59
12	Square			1410.8	35.06
13	Hexagonal			1487.3	31.54
14	Circular	1.5	1.6	1553.8	28.48
15	Square			1422.9	34,50
16	Hexagonal			1493.2	31.26

**Table 4:** Values of critical load for perforated and unperforated profiles, for four different composite lay-ups for chosen case.

Laminate Lay-Up	Symbol	Pcr [N]		Difference [%]
		Unperforated Profile	Perforated Profile	
[0/45/−45/90/0]s	P1	2172.4	1802.3	17.04
[45/−45/90/0]s	P2	3259.3	2761.5	15.27
[90/−45/45/0]s	P3	2183.3	1788.5	18.08
[90/0/90/0]s	P4	1590.6	1124.2	29.32

## Data Availability

Data are contained within the article.
